# Turning the Page on Pen-and-Paper Questionnaires: Combining Ecological Momentary Assessment and Computer Adaptive Testing to Transform Psychological Assessment in the 21st Century

**DOI:** 10.3389/fpsyg.2016.01933

**Published:** 2017-01-19

**Authors:** Chris J. Gibbons

**Affiliations:** ^1^Cambridge Centre for Health Services Research, University of CambridgeCambridge, UK; ^2^The Psychometrics Centre, Judge Business School, University of CambridgeCambridge, UK

**Keywords:** ecological momentary assessment, patient reported outcomes, computer adaptive testing, electronic assessment, item response theory, rasch analysis

## Abstract

The current paper describes new opportunities for patient-centred assessment methods which have come about by the increased adoption of affordable smart technologies in biopsychosocial research and medical care. In this commentary, we review modern assessment methods including item response theory (IRT), computer adaptive testing (CAT), and ecological momentary assessment (EMA) and explain how these methods may be combined to improve psychological assessment. We demonstrate both how a ‘naïve’ selection of a small group of items in an EMA can lead to unacceptably unreliable assessments and how IRT can provide detailed information on the individual information that each item gives thus allowing short form assessments to be selected with acceptable reliability. The combination of CAT and IRT can ensure assessments are precise, efficient, and well targeted to the individual; allowing EMAs to be both brief and accurate.

## Main Body

We are progressing through Information Age, the era whose roots took hold with the invention of the world-wide web and, driven by strong market forces, has yielded significant advances in computational power, information storage and retrieval, and the ability to access information across the globe. As more are more interactions are taking place online, so too are more data being collected from these interactions (Kosinski et al., 2013). More recently, there has been an explosion in the development, distribution, and use of techniques to make sense of the rapidly increasing volumes of data (Efron and Hastie, 2016).

Despite the near-ubiquity of internet-enabled devices and a significant increase in the number of human activities being mediated by digital products and services (Lambiotte and Kosinski, 2014), the advantages of this technological explosion have not been fully realized in many areas of psychology and medical research. One such example is psychological testing using psychometrically validated questionnaires, which is still largely dominated by a ‘pen-and-paper’ mindset which does not capitalize on many recent technological innovations. These techniques are limited by recall bias and may be liable to change over a short time course. While there has been some progress insofar as many psychometric assessments are now available in an electronic format, there has been little change in the way they are presented, scored, or used.

This paper discusses two notable exceptions where progress has been made, namely in the application of modern probabilistic psychometric techniques, including item response theory (IRT) and computer adaptive testing (CAT), as well as ecological momentary assessment (EMA). While modern psychometric techniques and EMA have brought forth significant advances in assessment techniques; they have done so largely in isolation from one another. This paper argues that these two techniques could be usefully integrated to drive psychological assessment further in a way that is both ecologically valid and, crucially, psychometrically accurate.

Ecological momentary assessment is the term used to describe some research methods which allows patients and participants to report on their experiences in real-time, in real-world settings, in multiple contexts, and repeatedly over time (Stone and Shiffman, 1994). Ecological momentary assessment can collect data using diverse modalities which can include diaries, open-text, and questions with Likert-type responses. It is common for such EMA platforms to be placed into apps which can be installed on mobile phones or tablets to facilitate responsive round-the-clock assessment. To reduce the burden naturally associated with multiple repeated assessments EMAs commonly include a small number of Likert-type questions or a reduced-length version of an existing questionnaire. This practice is often conducted with limited psychometric justification which may seriously affect the reliability of the scores derived from EMA assessments (Stone et al., 1994; Tasca et al., 2009; Palmier-Claus et al., 2013; Rosen and Factor, 2015).

While EMA offers a way to deal with the recall bias and natural variation that might affect the accuracy and interpretability of scores taken from a psychometric questionnaire it does not, on its own, offer any solution for ensuring that such assessments are reliable. In contrast, modern psychometric techniques, and especially CAT, can provide accurate and reliable estimates in reduced-length psychological evaluations. Computer adaptive testing refers to the use of algorithms which match questionnaire takers with the most relevant questions for them. The CAT process has been shown to increase measurement precision and efficiency greatly, allowing assessments to be shorter and more reliable than their paper-based fixed length counterparts (Gibbons et al., 2016).

Computer adaptive testing requires a calibrated ‘bank’ of items which contains information derived from modern psychometric models and methods which known as IRT (Van Der Linden and Glas, 2000; Wainer, 2000). Item response theory suggests that latent constructs vary in magnitude along a unidimensional linear continuum referred to as theta (θ). These theories explain how it is possible, using probabilistic estimation, to simultaneously model the level of underlying construct that a person has, and the level of the underlying trait that the item or questionnaire assesses (Hambleton et al., 1991). Mathematically, and in its simplest form, this can be expressed using Equation 1, shown below.

Equation 1: the Rasch model (Rasch, 1960), a one-parameter logistic IRT model

$$p_{i}\left(1|\theta \right)=\frac{e^{\left(\theta -b_{i}\right)}}{1+e^{\left(\theta -b_{i}\right)}}$$

Where 1 is a correct response (or, in the case of a psychological assessment a positive endorsement of the item), θ is the level of the underlying trait, *i* represents the items being answered and *b* represents the level of the trait necessary to have a 50 probability of endorsing the item (Rasch, 1960).

Item response theory has strict assumptions and produces assessments with robust measurement properties (Karabatsos, 2001). Their use in medical research was popularized over the past decade, at least in part, because of their ability to produce measures which could be shorter and more reliable than using classical test theory alternatives alone (Reeve et al., 2007; Gibbons et al., 2011).

The probabilistic underpinning of IRT allows it to simultaneously calibrate the level of the underlying construct which is measured by the individual items and the people responding to the assessment. The ability to calibrate each item independently of the overall scale is unique to IRT and means that evaluations can be made using subsets of items, rather than giving the entire questionnaire to each participant. Additionally, IRT can precisely calculate the measurement precision of any assessment regardless of the underlying level of the construct that the participant has or the number of items that they have completed. In contrast, classical test methods only give a single mean reliability value for the entire test, meaning that every item should be administered in each assessment to avoid unreliable estimates. The use of classical test methods also precludes the estimation the level of precision that evaluation using a subsample of items can give (Hays et al., 2000).

**FIGURE 1:**
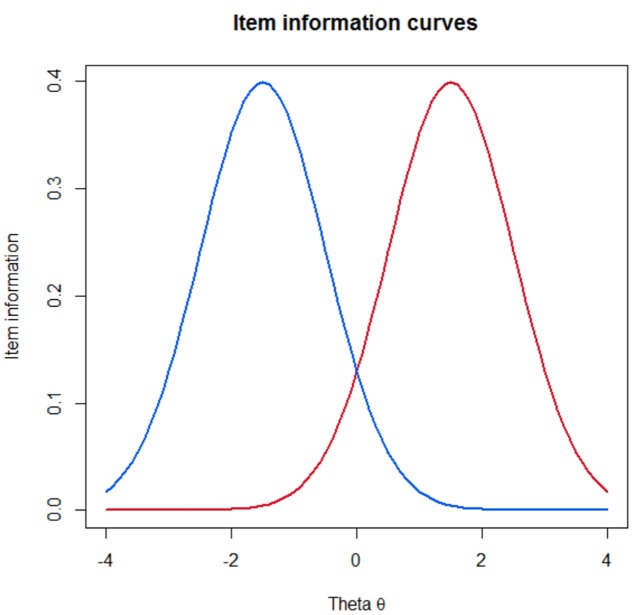
**Item information plots for two items which give good information at a high level of the underlying trait (theta) and at a low level**.

As well as being able to calculate the precision of a given assessment of any length using IRT methods once it has been given, it is also possible to assess the level of information that is available for each item individually. Knowing the information that every individual item gives allows researchers to pre-select a set of items which will give an adequate level of precision for a given assessment. **Figure 1** illustrate this principal and shows one item which gives a lot of information at a high degree of the underlying trait, and one which offers high information at a low level of the underlying trait.

Computer adaptive testing follows an iterative process of selecting items which are the most informative and suitable for the candidate taking the assessment, using both Bayesian and maximum information estimation methods (Magis and Raîche, 2011). By only administering items which reflect high information at the test taker’s level of the underlying construct, CAT achieves dual advantages of briefer assessments which consist only of the most relevant items (Gershon, 2005; Dosch, 2010).

Evaluation of individual item information across a scale would show the potential pitfalls regarding measurement accuracy if items were to be chosen without any consideration for their empirical psychometric qualities. Measurement accuracy can be expressed in terms of standard error, information, or reliability. The three are related as demonstrated in Equations 2–5.

Equation 2. Item information and standard error

$$SE\left(\theta \right)=\frac{1}{\sqrt{I\left(\theta \right)}}$$

Equation 3. Item information and assessment reliability

$$r\left(\theta \right)=1-{\left(\frac{1}{\sqrt{I\left(\theta \right)}}\right)}^{2}$$

Equation 4. Reliability and standard error

$$r\left(\theta \right)=1-SE{\left(\theta \right)}^{2}$$

**FIGURE 2:**
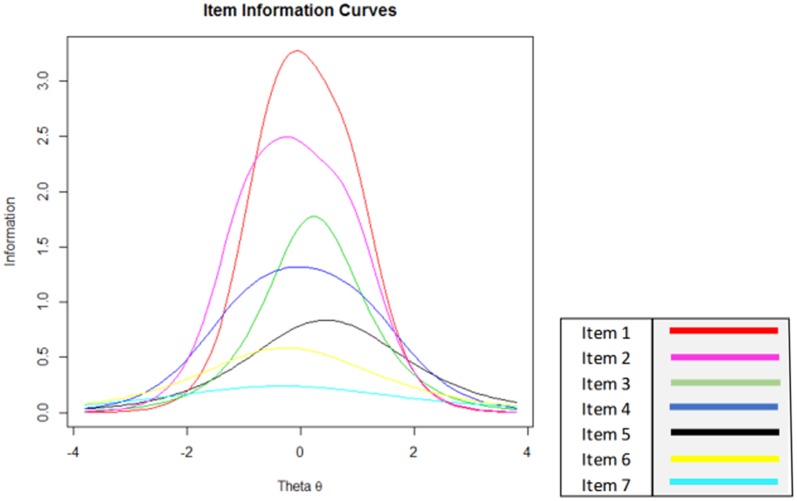
**Individual item information for a hypothetical assessment scale**.

Equation **Figure 2** shows item information for an entire scale consisting of seven items, in practice an item bank might be much larger than this. Using this figure, it is possible to see the risks associated with random selection of items in a reduced-length assessment.

A selection of three items to measure a person with an average level of the underlying trait or construct (theta = 0) can mean that assessment information ranges from 6.5 [using items 1, 2, and 3; equivalent to reliability = 0.85 (*SE* = 0.39)], and 1.5 [using items 5, 6, and 7; equivalent to reliability = 0.33 (*SE* = 0.82)]. The former would be regarded as an accurate assessment, whereas the reliability of the latter falls below any recommended level for individual or group assessments. It is also clear that in figure two that assessments for people with a very high or low theta value (greater than +2 or less than -2) will always necessitate more items before an acceptable level of information has been reached, highlighting both the need to take the individuals level of the underlying construct into account in each assessment and the risk of assuming that questionnaire items will yield uniform levels of information for each respondent. When the information in **Figure 2** is available to researchers who are interested in EMA testing, then a selection of items can be either hand-picked or delivered using a CAT protocol.

In addition to maximizing assessment precision whilst minimizing the number of items that a participant must respond to it is possible to programme CATs to behave in a way that further advantages their use within EMA. For example, it is possible to set simple logical rules which prevent the same item from being shown during consecutive assessments, limiting response biases caused by over-familiarity with items. A similar logical rule may also be used to ensure that certain important items are always asked; like questions relating to suicide, for example.

There is great potential to maximize the accuracy of psychometric assessments using EMA through the introduction of IRT and CAT methodologies. The combination of these techniques represents the most progressive thinking in terms of patient-reported assessment that allows assessments to be accurate, ecologically valid, and well targeted to the individual.

## Author Contributions

The author confirms being the sole contributor of this work and approved it for publication.

## Conflict of Interest Statement

The author declares that the research was conducted in the absence of any commercial or financial relationships that could be construed as a potential conflict of interest.
